# Three-Dimensional Analysis of Initial Brace Correction in the Setting of Adolescent Idiopathic Scoliosis

**DOI:** 10.3390/jcm8111804

**Published:** 2019-10-28

**Authors:** Haidara Almansour, Wojciech Pepke, Thomas Bruckner, Bassel G. Diebo, Michael Akbar

**Affiliations:** 1 Clinic for Orthopedics and Trauma Surgery, Heidelberg University Hospital, 69118 Heidelberg, Germany; haidar.almansour@gmail.com (H.A.); wojciech.pepke@med.uni-heidelberg.de (W.P.); 2 Institute of Medical Biometry and Informatics, University of Heidelberg, 69118 Heidelberg, Germany; bruckner@imbi.uni-heidelberg.de; 3 Department of Orthopedic Surgery, SUNY Downstate Medical Center, Brooklyn, New York, NY 11203, USA; dr.basseldiebo@gmail.com

**Keywords:** adolescent idiopathic scoliosis, 3D, Chêneau, brace, stereoradiography, Cobb angle, sagittal alignment, transverse plane parameters, axial rotation

## Abstract

The three-dimensional nature of adolescent idiopathic scoliosis (AIS) necessitates a tridimensional assessment and management. Bracing constitutes the mainstay conservative treatment for mild adolescent idiopathic scoliosis. In the literature hitherto, there has been uncertainty regarding the behavior of the spine, pelvis, and vertebral orientations in the context of bracing, especially in the transverse plane. This poses a challenge to healthcare providers, patients, and their families, as brace treatment, although not as invasive as surgery, is laden with medical and psychological complications and could be considered traumatizing. Hence, a thorough understanding of initial three-dimensional spinal behavior in the context of bracing is important. The purpose of this retrospective study was to investigate the immediate 3D impact of Chêneau-type brace. Thirty-eight patients with AIS undergoing Chêneau-type bracing were included. Patients were stratified according to their structural curve topography into thoracic, thoracolumbar, and lumbar groups. 3D reconstruction of the spine using a dedicated biplanar stereoradiography software with and without the brace was performed. The examined anthropometric radiographic measures were pre- to in-brace variations and differences of spinopelvic parameters and vertebral orientations in the coronal, sagittal, and transverse planes. The complex impact of the Chêneau-type brace on different curves in three planes was delineated. In the coronal plane, the Cobb angle was significantly decreased in all types of curves, and the coronal tilt correction was concentrated in specific segments. The impact of the brace in this study on the sagittal profile was variable, including the loss of thoracic kyphosis and lumbar lordosis. In the transverse plane, an axial vertebral rotation change and detorsion above the apex occurred in the thoracolumbar curves. The results from this exploratory study could shed some light on the initial 3D spinal behavior in the context of bracing and may be of beneficial for treating physicians and brace makers.

## 1. Introduction 

Adolescent idiopathic scoliosis (AIS) is a three-dimensional (3D) deformity of the spine and trunk. It affects 1%–3% of children aged 10–16 years [1]. The 3D nature of adolescent idiopathic scoliosis (AIS) necessitates a tridimensional assessment and management. 

Before Perdriolle et al. [2], AIS was a mere lateral curvature on the coronal plane [3]. The advent of new 3D imaging and postprocessing techniques triggered novel research to address the etiology, pathomechanism, diagnosis, and treatment of spinal deformities in the coronal, sagittal, and transverse planes. 

The treatment modalities of AIS are non-operative and surgical. Brace treatment is the cornerstone of non-operative management that aims to halt the progression of deformity before reaching the surgical threshold. In Europe, one of the most commonly used types of thoracolumbosacral orthoses is the Chêneau-type brace (C-brace) [4]. Using multiple pressure areas and expansion chambers, this brace intends to tackle the scoliosis in its three dimensions and regionally de-rotate the apical segment of the scoliotic curve [4]. Its efficacy in the coronal plane in halting curve progression has been established [5]. However, data on sagittal and axial correction remain sparse [6]. In terms of bracing biomechanics, few correction principles have reached final consensus among the International Society on Scoliosis Orthopaedic and Rehabilitation Treatment SOSORT expert committee [7].

This poses a challenge to healthcare providers and patients and their families, as brace treatment, although not as invasive as surgery, is laden with medical and psychological complications [8]. While medical complications such as tubular thorax and reduced total lung capacity due to prolonged wearing, reflux esophagitis due to elevated intragastric pressure, reduced glomerular filtration ratio due to compression, and pressure sores could be addressed with careful follow-up and adjustment of the brace, the psychological repercussions are far more difficult to handle [8,9]. These repercussions have an overreaching negative impact on the psychology of the treated children and their families [8,10]. It has been reported that even within the first month after the initiation of brace treatment, the rate of psychological problems rose from 7.6% to 82.1% [8,11]. The emotional distress caused by bracing treatment has been reported to be traumatizing and may have “lasting emotional scars” [12]. This in turn leads to a vicious circle of poor compliance on the basis of poorer vitality, psychological and social wellbeing, as well as decreased overall quality of life and inefficacy of brace treatment [12].

In light of the aforementioned serious implications, a deeper understanding of initial brace correction could be of interest, if not of significant importance, for the treating physician as well as the patient and their parents. This understanding is hindered by the lack of emphasis on the 3D correction of braces due to outdated imaging techniques. Indeed, pelvic morphology and coronal and sagittal spinal alignment are instrumental to the development and progression of adolescent idiopathic scoliosis [13,14]. However, transverse plane parameters such as axial rotation of the vertebrae have been shown to be significant risk factors for scoliotic curve progression [15]. The importance of a new 3D classification of scoliotic deformities has already been emphasized by the Scoliosis Research Society (SRS) 3D committee. They showed that two spines with similar 2D characteristics could have significantly varying 3D morphologies [15,16]. 

Initial in-brace 3D correction is important to predict the final treatment outcome [17,18]. The aim of this study was to investigate the immediate in-brace 3D impact of C-brace on the coronal, sagittal, and transverse planes. 

## 2. Materials and Methods 

### 2.1. Inclusion/Exclusion of the Patient Population 

This is a retrospective single-center study of patients with AIS between 2015 and 2017. The study was approved by the institutional review board committee (permission No. S-378).

**Figure 1:**
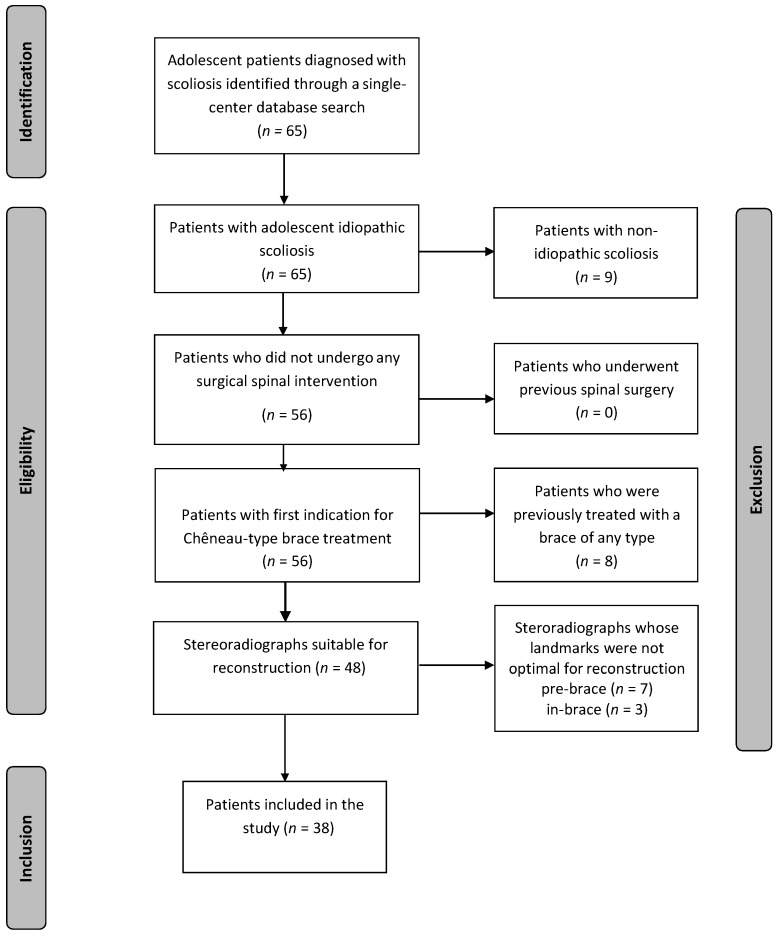
Flow diagram illustrating the inclusion/exclusion process of the study population.

Inclusion criteria were patients with AIS with Risser grade 0–2, a Cobb angle between 20° and 40°, a new prescription for a C-brace, aged >10 years [19], and for whom orthogonal full-spine stereoradiographs were obtained. Clinical and demographic characteristics were obtained from medical records. Exclusion criteria were patients with non-idiopathic scoliosis (like congenital, neuromuscular, or syndromic scoliosis), previous treatment with a brace of any type, antecedent spinal or pelvic surgical intervention, patients with no stereoradiographs, or images without visible femoral heads or T1 upper plate or inadequate delineation of their anatomic landmarks [20]. Figure 1 illustrates the inclusion and exclusion process. 

### 2.2. Radiographic Acquisition and Tridimensional Reconstruction

Radiographs were acquired using an EOS-imaging device, which is a low-dose biplanar imaging apparatus, providing simultaneous anterior-posterior and lateral views in a standing position (EOS-imaging^®^, Paris, France) [21]. Patients were barefoot, had their hands on their cheeks to avoid superimposition of the upper extremities on the spine [22], and were instructed to look straight ahead in a relaxed position. Radiographs that failed to fulfill these requirements were excluded. The average time between the pre-brace and in-brace radiographs was 4 months. Semiautomatic 3D reconstruction of the spine and pelvis by a single operator ensued via validated and dedicated software [23]. To counteract a potential pelvic axial rotation of during image acquisition, all parameters were expressed in the patients’ reference system based on a vertical plane traversing through the centers of the acetabulums as defined by SRS [22,24]. All measurements were performed by the first author (AH), a research fellow with a medical and biomedical engineering background who was not involved in the treatment of those patients nor in the production of the brace. 

### 2.3. Anthropometric Outcome Measures and Radiographic Analysis 

**Figure 2:**
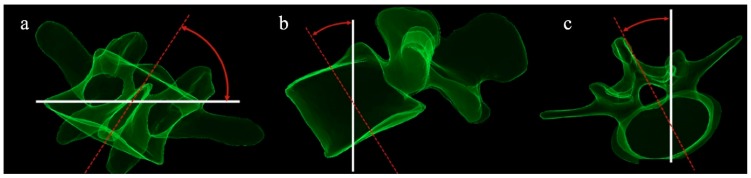
Vertebral rotations. (**a**) Coronal tilt; (**b**) sagittal tilt; (**c**) axial rotation.

Outcome measures were pre- to in-brace changes of vertebral and spinopelvic parameters in three planes. Radiographic parameters included the following: 

Coronal parameters: Cobb angle, coronal vertebral tilt (T1-L5) (Figure 2a), and pelvic obliquity.

Sagittal spinopelvic parameters: 

Regional parameters: thoracic kyphosis TK (T1–T12) and TK (T4–T12), lumbar lordosis (LL–L5) and LL-S1), sagittal vertebral tilt (T1–L5) (Figure 2b), pelvic incidence (PI), pelvic tilt (PT), and sacral slope (SS).

Global alignment: Sagittal vertical axis (SVA), *T*1—spinopelvic inclination (T1 SPi), *T*9—spinopelvic inclination (T9 SPi), and spinosacral angle (SSA).

**Figure 3:**
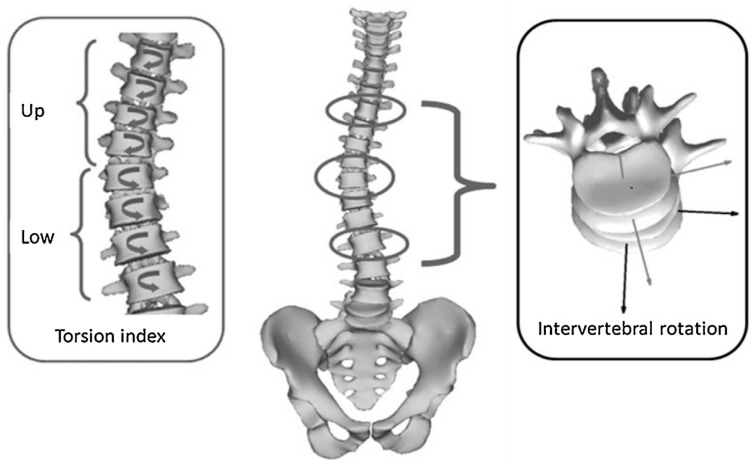
Transverse plane parameters including intervertebral rotation and torsion index. Reproduced from [22]. Copyright © 2015 Elsevier Masson SAS. All rights reserved.

Transverse plane parameters (Figure 3): apical vertebral rotation (AVR): axial rotation of the apical vertebra and axial vertebral rotation (T1–L5). Intervertebral axial rotation (IAR) corresponded to the axial rotation of the upper vertebra in the plane of the lower vertebra. IAR was calculated at the upper neutral zone (upper IAR) and the lower neutral zone (lower IAR) as described by Perdriolle et al. [2,22,25], and the torsion index (*Ti*) as described by Steib et al. (the mean of the two sums of IAR from the lower junction to the apex, and then from the apex to the upper junction) [25,26]. Subsequently, a “detorsion index” ((*Ti* pre brace − *Ti* in-brace)/*Ti* pre brace × 100) was computed to quantify the immediate rotational in-brace correction of torsion of the structural curve as a percentage [26].

### 2.4. Patient Stratification 

Patients were grouped according to their scoliotic curve topography into three groups: structural thoracic curves with an apex between T3 and T9, thoracolumbar curves with an apex between T10 and L1, and lumbar curves with an apex between L2 and L4.

### 2.5. Statistical Analysis

Descriptive statistics were reported as mean ± standard deviation (SD). Paired *t*-test was utilized to compute the pre- to in-brace changes. The threshold for statistical significance was set at 0.05. Absolute values of vertebral rotations (coronal tilt and axial rotation) were utilized to account for the severity of rotation without considering the direction of rotation. Software package SPSS 20.00 (IBM, Armonk, NY, USA) was used for statistical analysis.

## 3. Results 

### 3.1. Global Analysis

Thirty-eight patients (76% females) aged 12.6 ± 2 years (range 10–16 years) were included. Patients had a mean Cobb angle of 31.4 ± 6.4° range (19–41°). A total of 39% of patients had a Risser grade 0, 24% had a Risser grade 1, and 37% of included patients had a Risser grade 2. There were 19 patients with a structural thoracic curve with a mean Cobb angle 34.2 ± 5.6°, 9 patients with a structural thoracolumbar with a mean Cobb angle 29.4 ± 6.4°, and 10 patients with a structural lumbar curve with a mean Cobb angle of 27.6 ± 6.2°. In 90% (34 cases) of our patients, the Cobb angle decreased more than 5° and was unchanged (less than 5°) in 10% (4 cases). In 61% of patients (23 cases), Cobb decreased more than 10°. Regarding transverse plane parameters, in 32% (12 cases) of our patients, AVR decreased more than 5°, remained unchanged in 58% (22 cases), and increased more than 5° in 10% (4 cases) of the study population.

In terms of sagittal alignment, there was a statistically significant, although modest, mean decrease of TK T4–T12 by 2.6° (Table 1). Similarly, there was a mean statistically significant modest decrease of LL–L5 by 2.6° and of LL–S1 by 3.8°. Regarding global sagittal alignment, global comparison revealed a statistically significant decrease of SSA by 2.4°. In terms of pelvic parameters, global pre- to in-brace comparison revealed a statistically significant flattening of SS by 3.3°. 

Table 1 elucidates the global pre- to in-brace changes of sagittal spinopelvic parameters regardless of curve type.

### 3.2. Sub-Analysis by Curve Type

#### 3.2.1. Coronal Plane

A significant decrease of the mean Cobb angle was noted by 10.6 ± 6.0° (*p* = 0.0001) for the thoracic curves, by 16.5 ± 5.5° (*p* = 0.0001) for the thoracolumbar curves, and by 12.4 ± 6.7° (*p* = 0.0001) for the lumbar curves. Pre- to in-brace changes of coronal, sagittal, and transverse plane parameters according to curve type are elucidated in Table 2.

**Figure 4:**
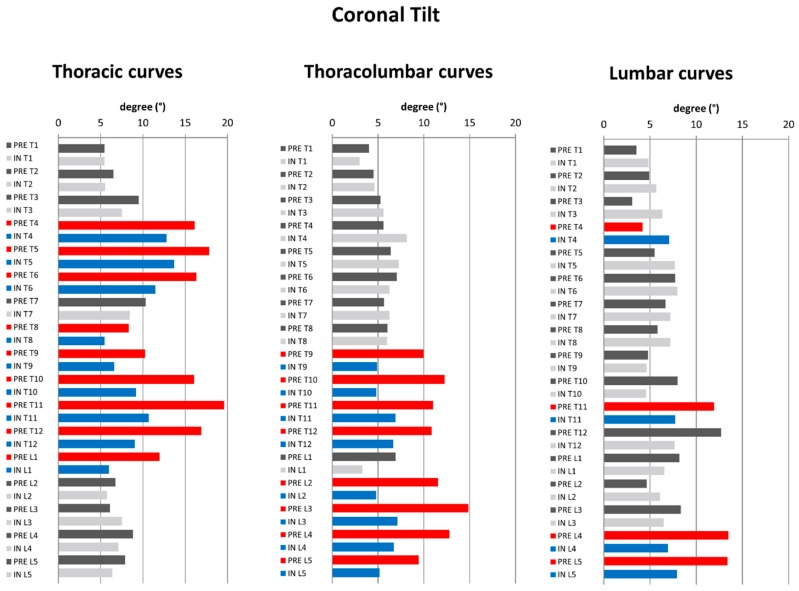
Diagrams illustrating the pre- to in-brace changes in vertebral coronal tilt (T1–L5) pertaining to structural curve type. Dark grey: pre-brace, light grey: in-brace. Statistically significant changes in pre- to in–brace vertebral orientations are depicted in red (pre-brace) and blue (in–brace) (*p* ≤ 0.05).

In terms of coronal vertebral tilt (Figure 2a), thoracic curves revealed a significant decrease of T4–L1 coronal tilt with the exception of T7. Thoracolumbar curves revealed a significant decrease of T9–L5 with the exception of L1. Lumbar curves revealed a significant decrease of L4–L5 coronal vertebral tilt (Figure 4).

#### 3.2.2. Regional and Global Sagittal Profile 

**Figure 5:**
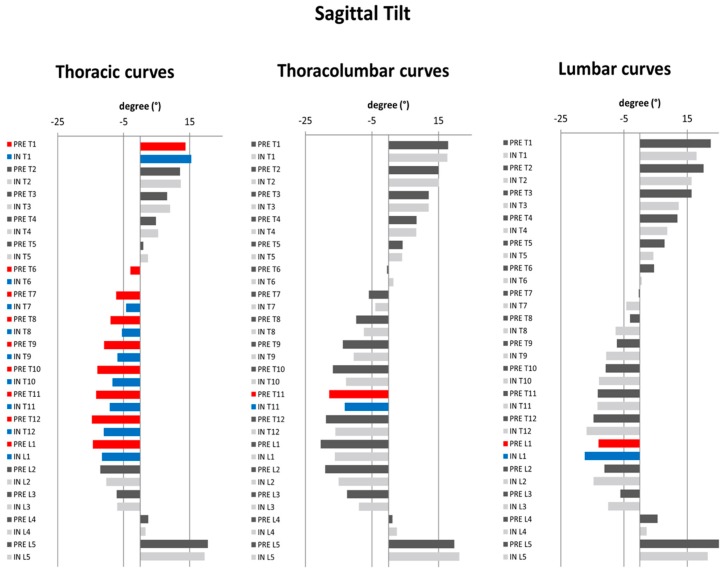
Diagrams illustrating the pre- to in-brace changes in vertebral sagittal tilt (T1–L5) pertaining to structural curve type. Dark grey: pre-brace, light grey: in-brace. Statistically significant changes in pre- to in-brace vertebral orientations are depicted in red (pre-brace) and blue (in-brace) (*p* ≤ 0.05).

In thoracic curves, a statistically significant mean decrease of TK (T4/T12) by 3.1 ± 5.3° (*p* = 0.021) was observed. Similarly, a statistically significant mean diminution of LL L1/L5 by 4.1 ± 5.7° (*p* = 0.006), and of LL L1/S1 by 5.4 ± 5.6° (*p* = 0.001) was observed. SVA had a statistically significant increase of 11.2 ± 20.7mm (*p* = 0.03). SSA showed a decrease of 4.4 ± 5.1° (*p* = 0.001). SS was the only pelvic parameter which revealed statistically significant mean decrease of 4° ± 6.2° (*p* = 0.011) (Table 2). In thoracolumbar curves, a statistically significant mean diminution of LL L1/S1 by 7.5 ± 8.6° (*p* = 0.032) was revealed. A statistically significant mean pre- to in–brace diminution of SSA by 4.1 ± 3.5° was observed (*p* = 0.007) (Table 2). In lumbar curves, no change of sagittal spinopelvic parameters was observed, with the exception of SVA, which exhibited a mean posterior shift 24.8 ± 28.9 mm (*p* = 0.024), and T1Spi, which showed a modest increase of 2.9 ± 3.7° (*p* = 0.036). In terms of sagittal vertebral tilt (Figure 2b), thoracic curves revealed a significant increase of T1 and decrease of T6–L1 sagittal vertebral tilt. For thoracolumbar and lumbar curves, there was a significant, though clinically irrelevant change of T11 and T9, respectively (Figure 5). 

#### 3.2.3. Transverse Plane Parameters (Figure 3)

No statistically significant changes for thoracic and lumbar curves could be noted. However, the only statistically significant pre- to in-brace mean changes were noted in the thoracolumbar curves; AVR (4.4 ± 4.2° (30% de-rotation) (*p* = 0.014)), torsion index (3 ± 2.9° (43% detorsion) (*p* = 0.016)), and upper IAR (3.8 ± 3.8° (48% de-rotation) (*p* = 0.018)) (Table 2). 

**Figure 6:**
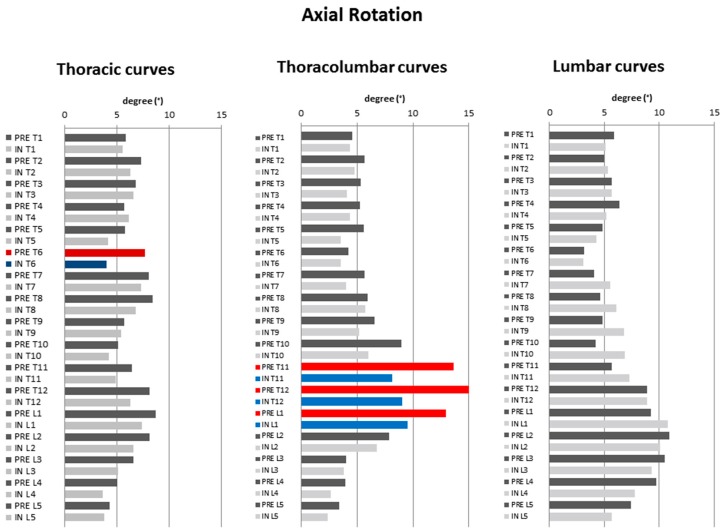
Diagrams illustrating the pre- to in-brace changes in vertebral axial rotation pertaining to structural curve type. Dark grey: pre-brace, light grey: in–brace. Statistically significant changes in pre- to in-brace vertebral orientations are depicted in red (pre-brace) and blue (in-brace) (*p* ≤ 0.05).

In terms of T1–L5 axial vertebral rotation (Figure 2c), for thoracic curves, the only statistically significant difference could be observed in T6. For thoracolumbar curves, a statistically significant decrease of axial rotation was noted in T11–L1. For lumbar curves, no statistically significant differences could be observed (Figure 6).

## 4. Discussion 

The construction of a brace resembles a complex engineering act entailing a multitude of decisions that could have a direct impact on the final treatment outcome. The C-brace should not be regarded as an “orthopaedic product“; it should be considered as an evolving concept and a dynamic continuum in the quest to achieve the highest treatment standards and patient safety [18]. A well-delineated concept that was associated with a successful bracing outcome is the short-term in-brace correction [17,18]. However, few correction principles reached final consensus among the SOSORT expert committee [7]. Research on brace treatment is in the midst of a paradigm shift regarding the conceptualization of 3D correction methods which triggered the interest to delve deeper into the realm of 3D brace analysis. 

The global analysis of this study showed that Chêneau type in-brace changes of 3D spinal alignment occur. Cobb correction in the present study revealed similar results to the correction noted by the literature [4,27]. Coronally, the Cobb angle was significantly decreased in all types of curves. In most patients (90%), it was reduced by more than 5°. Fang et al. showed that in their patient series, a similar correction was noted but was much more variable, with half of the patients experiencing a decrease of more than 5°, 31% remaining unchanged, and an increase of more than 5° in 19%. Courvoisier et al. revealed a correction of the Cobb in 50% and stability of the Cobb in 50% [4,27]. 

On the other hand, to our knowledge, literature on segmental coronal tilt is very sparse. Coronal tilt correction was concentrated in specific segments: Thoracic curves revealed a significant decrease of T4–T6 and T8–L1, thoracolumbar curves T9–T12 and L2–L5, and lumbar curves L4–L5. (Figure 4). 

**Figure 7:**
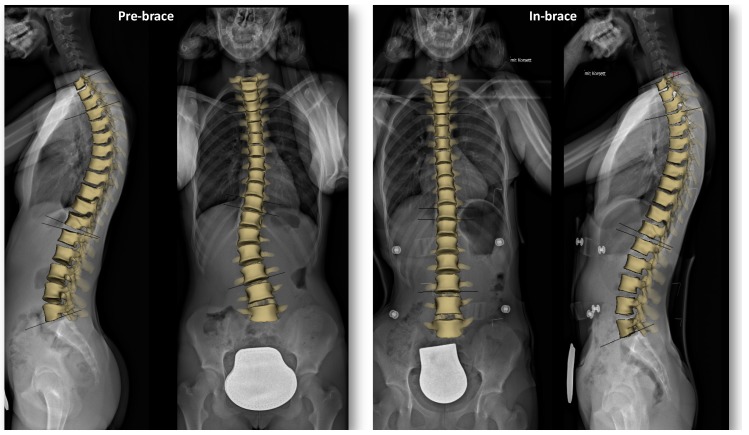
A 3D spinal reconstruction superimposed on biplanar stereoradiographs for a 14-year-old patient undergoing bracing treatment. This figure depicts the coronal correction of the thoracolumbar scoliotic curve.

This could be explained by the morphology of scoliosis and biomechanics of the C-brace. At the apex, the vertebrae are more horizontally aligned and hence have less coronal tilting. Analyzing the apices of the included curves, for the thoracic curves, the apex was mostly between T7 and T9, for thoracolumbar curve L1, and for lumbar curve L2. As a result, the cephalad and caudal vertebrae of the aforementioned apices revealed the greatest coronal tilt correction. Donzelli et al. analyzed the 3D correction of the Sforezco brace with an EOS system on 16 patients with AIS. Authors observed that coronal vertebral tilting was also segmentally concentrated (T3–T6 and T10–L1). However, their finding was global and was not curve-specific [28]. The Cobb angle alone is not enough to describe coronal correction. The measurement of segmental coronal tilting enables the characterization of the exact segmental coronal correction along the scoliotic curve and could allow a better identification of the quality of coronal correction. It is of note that the clinical relevance of coronal tilting remains to be validated. Figure 7 exemplarily illustrates coronal curve correction of one of the included patients. 

### 4.1. In-Brace Spinopelvic Sagittal Changes 

A secondary purpose of bracing treatment is to preserve the sagittal profile of the spine, and its effect should be viewed through a wider lens, as the multifaceted geometry of the sagittal profile cannot be assessed via simple angular correction [18,29]. The impact of the C-brace on the sagittal profile in this study was variable, which corroborates previous findings [4,25]. Regardless of the curve type, the flattening of regional curves such as TK and LL was noted. However, a trend could be delineated when curve-specific grouping was applied; most sagittal changes occurred in patients with thoracic curves. 

A significant loss of TK (T4–T12) was observed without the loss of TK T1–T12. Similarly, Donzelli et al. observed that TK (T4–T12) revealed a slight decreasing trend during treatment, while there were no changes in TK (T1–T12) [28]. The positioning of the thoracic posterior pad that pushes against the rib hump with the intention to regionally de-rotate the apical vertebra could be the reason for this iatrogenic loss of TK T4–T12. This effect has been previously shown [25,30]. Regarding the stability of TK T1–T12, this phenomenon could be explained by the segmental flexibility of the malleable spine of young patients. A potential compensation mechanism in the context of the brace-induced loss of TK T4–T12 might be a segmental sagittal tilt increase over the level of T4. This was evident in the statistically significant pre- to in-brace increase of T1 sagittal tilt (anterior tilt) (Figure 5)

The loss of lordosis was previously described by authors to occur in 63% and even 90% of the analyzed patient population [4,25,30]. In the literature hitherto, there was no correlation found between the diminution of LL and coronal correction. Therefore, this phenomenon was described as a “side effect” of bracing treatment in which the trunk follows brace shape [25,30]. Furthermore, it is important to note that lumbar and thoracic spine geometries should be globally analyzed due to their interrelation. The described hypokyphosis could drive a concomitant hypolordosis to safeguard sagittal alignment as a form of compensation and possibly maintain a minimum energy expenditure [31]. Despite the decrease of LL, the only pelvic change was a flattening of SS, while PT, the parameter which indicates pelvic compensation, remained unchanged. Indeed, the statistically significant change in LL could be considered too small to induce a relevant impact on the pelvis, which could explain why the PT has not changed.

In surgically treated patients, when thoracic kyphosis decreases, the lumbar spine compensates by decreasing lordosis to maintain the thoracolumbar harmony [32,33]. This interplay could also be translated to bracing treatment.

In terms of global alignment, SVA changed, showing anterior translation with the in-brace patients being more anteriorly-aligned; however, all patients retained acceptable sagittal alignment, as previously reported [34]. Moreover, a significant decrease in SSA was observed. SSA quantifies sagittal alignment and global kyphosis of the spine and depends on the location of C7 and sacral slope [29,31]. This decrease in SSA is a product of the anterior translation of SVA and flattening of SS. This would indicate that the C-brace has increased the global kyphosis of patients with thoracic scoliotic curves.

In summary, in-brace changes of the thoracic curves revealed a loss of TK T4–T12, a loss of LL, anteriorly aligned SVA, increased global kyphosis, and no employment of pelvic compensatory mechanisms. Correspondingly, anterior sagittal tilting occurred in the middle of the thoracic spine and thoracolumbar junction (T6–L1).

In terms of thoracolumbar curves, the only relevant change was the loss of LL and a decrease of SSA. The sagittal vertebral tilt revealed an increasing (anterior tilt) trend without reaching the threshold for statistical significance. This could be explained by the fact that SVA and pelvic parameters did not undergo statistically significant changes. In order to further understand lumbar geometry, LL should not be considered a solitary curve and should be segmentally analyzed [31]. In the present study, a distribution of the change in LL occurred over the five lumbar vertebrae, and hence, the change in sagittal vertebral tilting may have been too small to reach statistical significance. The decrease of SSA in this group could be explained by the accumulation of two factors, anterior alignment of SVA and the flattening of SS. However, both were statistically insignificant and could have led to the statistically significant decrease of SSA. 

The lack of change for most of sagittal parameters (TK and LL) in the lumbar curves could be related to the brace’s characteristics, which are specifically designed to correct lumbar scoliotic curves. In our institution, lumbar C-braces are designed to be shorter than thoracolumbar and thoracic braces and exhibit pressure on the apex of the scoliotic spine laterally. Hence, no direct synthetic alteration of the sagittal profile could be noted. 

### 4.2. In-Brace Behavior of Transverse Plane Parameters

**Figure 8:**
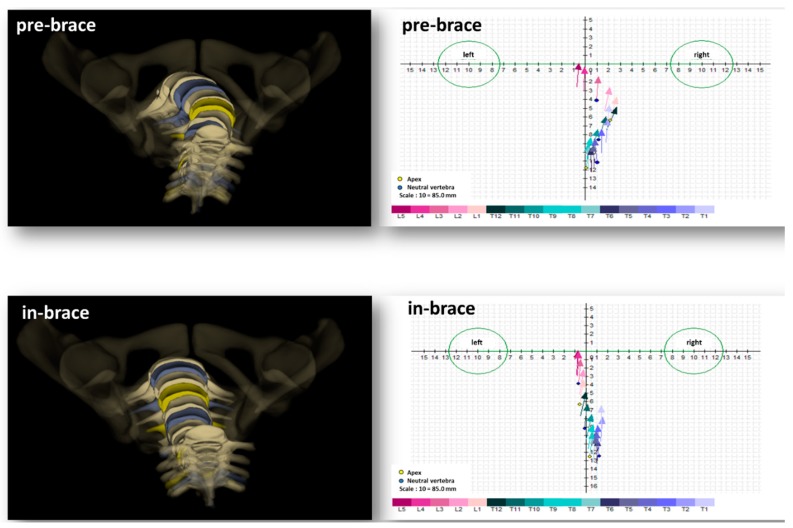
Transverse plane visualizations of the scoliotic spine of the same patient. This figure depicts the in-brace transverse plane changes of a structural thoracolumbar curve (apex L1). The **left upper** and **lower** panels illustrate pre- and in-brace 3D reconstructions. The **right upper** and **lower** panels illustrate a “top view” of the spine using the vector method as reported by Illes et al. [36]. The two spheres represent the right and left acetabulums. Each vector illustrates the axial rotation of T1–L5.

Achieving holistic correction and stabilization of the scoliotic curve is the impetus to analyze the transverse plane parameters in the setting of AIS [18]. Furthermore, it is now well-documented that there is a deficient understanding of the behavior of these parameters in the context of bracing treatment [25,35]. Figure 8 exemplarily illustrates the correction of AVR and depicts the rotation of each vertebra in the transverse planes. 

Studying these parameters could answer the question of whether bracing treatment is efficient in de-rotating a scoliotic spine. Moreover, it raises the question of whether this potential de-rotation occurs en bloc or with junctional intervertebral detorsion.

Our findings show that an axial vertebral change and detorsion occurred in the thoracolumbar curves (43% detorsion). This detorsion seems to have occurred from the upper junctional intervertebral de-rotation (48% de-rotation)-i.e., de-rotation ensues above the apex. This is in partial concordance with Donzelli et al., who observed a significant change of the torsion index in the thoracolumbar curves of their included 16 patients with AIS. Moreover, they observed a significant segmentally concentrated change of axial vertebral rotation in T10–L1 [28] (Figure 6). 

On the other hand, this is in contrast with the findings of Lebel et al., who analyzed a 3D C-brace in 18 females with AIS and showed that C-brace was able to reduce rotation of the apical vertebra by a delta of 8°. However, no significant junctional vertebral correction could be observed [37]. Similarly, Courvoisier et al. observed an en bloc de-rotation of the spine without influencing the junctional intervertebral rotation evident by alteration of the AVR without modification of the torsion [25]. It is important to note that different braces from different institutions and different curve morphologies were analyzed in the aforementioned studies, likely due to the small number of included patients. Moreover, the authors highlighted the high in-brace variability of the transverse plane parameters in their investigation of 30 patients. This variability is also evident in our results. Comparable to Courvoisier et al. [25], a worsening of AVR was noted, which again shows the lack of understanding of 3D correction methods. 

This study facilitates a deeper understanding of the mechanics of the C-brace correction and offers a detailed delineation of the intricacies of the C-brace impact on the scoliotic spine in three planes. A strength of this study is that one brace type was used from the same experienced brace maker which should decrease the bias related to the mixing of different brace designs from different institutions [6].

### 4.3. Limitations

Careful interpretation of these exploratory results is warranted. The generalizability of the results is limited by the study’s retrospective design and small sample size. Nevertheless, our sample size is asymptotic to many of the previous studies analyzing the brace effect in 3D. Indeed, some statistically significant changes, such as the ones of the sagittal profile do not necessarily reflect their clinical relevance. On one hand, the clinical relevance of these small changes might be reflected in the fact that the sagittal profile was safeguarded in the context of bracing [18]. On the other hand, the true clinical relevance of these minute changes must be validated, especially because they might fall within the error of measurement. Similar to the results of the sagittal profile, most changes in the transverse plane parameters and vertebral orientations could be regarded as too small to be truly considered clinically relevant; hence, a critical appraisal of their clinical value is warranted. Further, the clinical value of the “detorsion” and “torsion index” has not been shown [26]. Similarly, the lack of patient-reported outcomes constitutes an important limitation of this study.

Due to the small sample size, grouping based on Lenke classification was not possible. Furthermore, immediate in-brace correction does not directly translate into complete brace effectiveness. Our data cannot compete with those offered by longitudinal follow-up studies which assess post-weaning results and analyze curve progression [37]. Simultaneously, the lack of in-brace correction data has been discussed as a major limitation to brace studies [3]. Henceforth, our results may prove useful in covering this gap. The inherent heterogeneity of the brace effect could also be considered as a limiting factor [3]. This is reflected by the variability and large standard deviation for some of the discussed parameters. This variability is well documented and is attributed to multiple factors, such as the use of “global statistics” and comparing different curve topologies and different braces [3,4]. The presented study attempted to reduce these factors where possible. An important limitation of our study is its selection bias, which endangers the external validity of our results. This could be seen in the baseline data of the thoracolumbar curves, which had a larger pre-brace axial vertebral rotation. This hinders the valid comparison of the C-brace effect on the thoracic, thoracolumbar, and lumbar curves regarding transverse plane correction. Further investigations with a larger sample size are needed to characterize the transverse plane pattern of thoracic, thoracolumbar, and lumbar curves. Moreover, the brace maker’s experience and craft are vital to achieving good results. It could be a strong point that all patients were treated by the senior author using only one brace type fabricated by an experienced brace maker [6]. However, we concede this to be a limitation. The craft of the brace maker is pivotal to enhancing the efficacy of bracing and hence would bias the discussed results [4,25]. Simultaneously, this represents one of the main motivations to computerize, “parametrize”, and exclude the “craft part” of the brace production process using baseline data of the in-brace correction, such as the present study [3]. Finally, the measurement error from using the software should be considered. Though the reliability of the 3D measurements is very good, a previous reliability study [20] demonstrated that the measurement errors of the discussed parameters could be as high as 3.5 degrees. 

## 5. Conclusions

This retrospective pre- to in-brace analysis delineated the complex impact of the C-brace on different curves in 3D. In summary, coronally, the Cobb angle was significantly decreased in all types of curves, and coronal tilt correction was concentrated in specific segments. The impact of the C-brace in this study on the sagittal profile was variable, including the loss of TK and LL. Most sagittal changes were observed in patients with thoracic structural curves. In the transverse planes, an axial vertebral correction and detorsion occurred in the thoracolumbar curves (43% detorsion). Results from this exploratory study could shed some light on initial 3D spinal behavior in the context of bracing and may be of beneficence for treating physicians and brace makers. It is important to note that most changes were very small and that statistical significance and clinical relevance in the context of a small sample size and lack of patient-reported outcomes are not interchangeable.

## Figures and Tables

**Table 1:** Pre- to in-brace changes of sagittal parameters regardless of curve type.

Global Changes of Sagittal Parameter					
	Before Brace	In Brace	Mean Difference	*p*	Absolute Mean Difference
**TK (T1/T12)**	33.2 ± 11.5	30.9 ± 11.3	2.3 ± 8.3	0.095	2.3 ± 8.3
**TK (T4/T12)**	24.8 ± 12.6	22.2 ± 12.4	2.6 ± 6.3	**0.017**	2.6 ± 6.3
**LL (L1/L5)**	43.9 ± 10.4	41.3 ± 11.0	2.6 ± 6.7	**0.020**	2.6 ± 6.7
**LL (L1/S1)**	55.7 ± 10.8	52.0 ± 12.1	3.8 ± 7.5	**0.004**	3.8 ± 7.5
**PT**	6.7 ± 8.0	8.3 ± 7.8	−1.6 ± 4.9	0.057	3.6 ± 3.7
**PI**	48.3 ± 12.0	46.6 ± 12.8	1.7 ± 6.3	0.109	1.7 ± 6.3
**SS**	41.6 ± 9.6	38.3 ± 10.0	3.3 ± 6.5	**0.004**	3.3 ± 6.5
**Pelvic Obliquity (mm)**	4.4 ± 2.7	4.1 ± 3.1	0.3 ± 2.5	0.446	0.3 ± 2.5
**SVA (mm)**	−8.3 ± 28.8	−6.7 ± 25.1	−1.6 ± 31.6	0.752	23.6 ± 20.6
**SSA**	132.7 ± 9.8	130.2 ± 9.7	2.4 ± 6.2	**0.019**	2.4 ± 6.2
**T1SPi**	4.0 ± 3.9	4.0 ± 3.6	−0.06 ± 4.0	0.921	3 ± 2.6
**T9SPi**	7.4 ± 5.0	7.3 ± 4.5	0.07 ± 4.7	0.930	3.4 ± 3.3
TK (TK1/TK12) = thoracic kyphosis measured from TK 1 to TK 12, TK (TK4/TK12) = thoracic kyphosis measured from TK 4 to TK 12, LL-L5 (L1/L5) = lumbar lordosis measured from L1 to L5, LL-S1 (L1/S1) = lumbar lordosis measured from L1 to S1, PT = pelvic tilt, PI = Pelvic incidence, SS = sacral slope, SVA = sagittal vertical axis (mm), SSA = spinosacral angle, T1SPi = T1 spinopelvic inclination, T9SPi = T9 spinopelvic inclination. Bold denotes statistical significance. All angles are given with °.					

**Table 2:** Correction of coronal, sagittal and transverse radiographic parameters with the Chêneau-type brace.

Stratification by Scoliosis Type												
	Thoracic				Thoracolumbar				Lumbar			
	Before Brace	In Brace	Mean Difference	*p*	Before Brace	In Brace	Mean Difference	*p*	Before Brace	In Brace	Mean Difference	*p*
**Cobb angle**	34.2 ± 5.6	23.5 ± 8.0	10.6 ± 6.0	**0.000**	29.4 ± 6.4	12.9 ± 8.1	16.5 ± 5.5	**0.000**	27.6 ± 6.2	15.6 ± 4.3	12.4 ± 6.7	**0.000**
**TK (T1/T12)**	29.0 ± 11.6	27.0 ± 11.1	1.9 ± 6.3	0.196	37.8 ± 7.4	33.8 ± 13.4	4.0 ± 10.3	0.276	36.9 ± 12.2	35.5 ± 7.6	1.5 ± 10.1	0.660
**TK (T4/T12)**	20.8 ± 14.5	17.7 ± 12.1	3.1 ± 5.3	**0.021**	29.3 ± 8.4	26.2 ± 13.2	3.2 ± 7.8	0.261	28.4 ± 9.7	27.4 ± 9.5	1.0 ± 7.2	0.658
**LL–L5 (L1/L5)**	42.2 ± 10.8	38.1 ± 12.0	4.1 ± 5.7	**0.006**	46.2 ± 7.4	42.7 ± 9.3	3.5 ± 7.2	0.183	45.0 ± 12.2	46.0 ± 9.4	−0.1 ± 7.2	0.672
**LL–S1 (L1/S1)**	53.0 ± 10.4	47.6 ± 12.6	5.4 ± 5.6	**0.001**	59.6 ± 9.5	52.2 ± 9.8	7.5 ± 8.6	**0.032**	57.3 ± 12.2	60.1 ± 8.9	−2.7 ± 6.0	0.186
**PT**	7.3 ± 8.7	8.4 ± 8.6	–1.2 ± 4.4	0.256	4.3 ± 7.2	5.8 ± 5.0	−1.5 ± 5.6	0.450	8.2 ± 7.8	10.0 ± 7.9	−1.8 ± 4.3	0.211
**PI**	47.3 ± 12.6	44.5 ± 14.1	2.8 ± 8.0	0.142	44.7 ± 7.8	43.6 ± 8.6	1.1 ± 4.1	0.433	53.3 ± 13.0	53.5 ± 12.3	−0.2 ± 4.0	0.882
**SS**	40.0 ± 10.7	36.0 ± 11.3	4.0 ± 6.2	**0.011**	40.4 ± 6.3	37.8 ± 6.7	2.6 ± 6.4	0.255	45.2 ± 9.5	43.6 ± 8.8	1.7 ± 5.7	0.379
**Pelvic Obliquity (mm)**	4.0 ± 2.3	4.3 ± 3.2	−0.4 ± 2.7	0.530	5.2 ± 2.7	4.4 ± 3.0	0.8 ± 2.3	0.336	4.8 ± 3.5	3.8 ± 3.2	1.0 ± 2.5	0.224
**SVA (mm)**	−11.1 ± 15.3	0.1 ± 22.7	−11.2 ± 20.7	**0.030**	−28.1 ± 29.5	−17.6 ± 32.2	−10.6 ± 39.0	0.441	15.0 ± 34.1	−9.9 ± 28.9	24.8 ± 28.9	**0.024**
**SSA**	132.3 ± 10.8	127.9 ± 11.2	4.4 ± 5.1	**0.001**	134.0 ± 8.1	129.9 ± 7.6	4.1 ± 3.5	**0.007**	132.1 ± 9.9	134.9 ± 7.2	−2.8 ± 7.2	0.249
**T1SPi**	4.3 ± 2.6	3.5 ± 3.6	0.7 ± 2.8	0.266	5.9 ± 4.0	4.4 ± 3.2	1.5 ± 5.3	0.432	1.7 ± 4.9	4.6 ± 4.0	−2.9 ± 3.7	**0.036**
**T9SPi**	6.9 ± 4.8	6.5 ± 5.1	0.4 ± 3.1	0.556	10.0 ± 4.6	7.8 ± 3.9	2.2 ± 6.6	0.351	5.9 ± 5.2	8.4 ± 3.6	−2.5 ± 4.8	0.135
**AVR**	8.1 ± 5.2	7.4 ± 4.4	0.7 ± 5.1	0.586	14.5 ± 8.6	10.2 ± 7.0	4.4 ± 4.2	**0.014**	10.6 ± 4.4	10.1 ± 4.2	0.6 ± 6.9	0.796
**Upper IAR**	8 ± 6.7	6.3 ± 4.7	1.7 ± 5.4	0.188	7.9 ± 4.3	4.1 ± 3.5	3.8 ± 3.8	**0.018**	5.6 ± 4.7	4.4 ± 3.8	1.3 ± 2.8	0.186
**Lower IAR**	7.4 ± 6.6	8.4 ± 5.2	0.9 ± 5.5	0.471	5.9 ± 3.9	3.7 ± 3.2	2.2 ± 3.8	0.117	4.4 ± 3.1	3.5 ± 2.9	0.8 ± 2.7	0.383
**Ti**	7.7 ± 6.1	7.3 ± 4.3	0.4 ± 5.1	0.744	6.9 ± 3.0	3.9 ± 3.2	3.0 ± 2.9	**0.016**	5.0 ± 2.6	3.9 ± 1.6	1.0 ± 1.7	0.092
TK (TK1/TK12) = thoracic kyphosis measured from TK 1 to TK 12, TK (TK4/TK12) = thoracic kyphosis measured from TK 4 to TK 12, LL (L1/L5) = lumbar lordosis measured from L1 to L5, LL (L1/S1) = lumbar lordosis measured from L1 to S1, PT = pelvic tilt, PI = pelvic incidence, SS = sacral slope, SVA = sagittal vertical axis (mm), SSA = spinosacral angle, T1SPi = T1 spinopelvic inclination, T9SPi = T9 spinopelvic inclination; AVR = axial rotation of the apical vertebra; IAR = intervertebral axial rotation; *Ti* = torsion index. Data expressed as mean ± standard deviation; **Bold** denotes statistical significance, *p* ≤ 0.05.												
